# Sale of Diplomas

**Published:** 1870-04

**Authors:** 


### Editorial.

### *Sale of Diplomas.*

About all the space that can be spared has been given to this subject. The Philadelphia University, through its Dean, W. Paine, M.D., expressly denies having been engaged in the business, but states that it is evidently the work of other parties. All the agencies hail from Philadelphia, and we are told there is a "Columbian University" in that city, and also a University of America, or American University, which operates in the same stock.

Meanwhile we can mention two practitioners in this city who have received diplomas recently from Philadelphia by direct purchase, neither of which is from the Columbian or American. We can also mention an honorary degree sent to a person here, who is not in practice, with an intimation that an honorarium of $50 was expected therefor.

To one of the first two alluded to was sent a private letter, which, taken in connection with the rest of the transaction, to our mind proves that the writer is or was a party to the business of diploma selling, and the name signed is that of a Professor in a Philadelphia "University," neither "Columbian" nor "American."

We do not propose to figure as parties in a threatened libel suit, as did he of the *Reporter*, or else we should give the names of all concerned, and publish the letter. Diploma selling has grown into a business as well conducted as counterfeiting.

